# Heat-Related Illnesses in India: Focus on Fluid-Electrolyte Management

**DOI:** 10.7759/cureus.117265

**Published:** 2026-09-30

**Authors:** Sanjay Kalra, L. Sreenivasa Murthy, Janani Shankar, Amol Patil, Harshad Malve, Vijay Chamle, Nikhil Bangale

**Affiliations:** 1 Endocrinology, Bharti Hospital, Karnal, IND; 2 Medicine, Life Care Hospital and Research Centre, Bengaluru, IND; 3 Pediatrics, Kanchi Kamakoti CHILDS Trust Hospital, Chennai, IND; 4 Medical Affairs, Kenvue, Mumbai, IND; 5 Medical Safety Sciences, JNTL Consumer Health (India) Pvt Ltd, Mumbai, IND; 6 Medical Safety Sciences, Kenvue, Mumbai, IND

**Keywords:** dehydration, dehydration management, electrolyte drinks, heat exhaustion, oral rehydration

## Abstract

Heat exhaustion represents a growing clinical and public health challenge, driven by escalating environmental heat exposure. Disturbances in body fluid homeostasis and electrolyte balance play a central role in its development, often resulting from prolonged heat stress and insufficient fluid replenishment. This narrative review synthesizes current evidence on the underlying mechanisms, symptom spectrum, and contemporary management approaches, with particular emphasis on hydration-based interventions.

The clinical profile spans early nonspecific symptoms to more pronounced physiological compromise, underscoring the importance of timely identification and intervention to prevent deterioration. Management priorities include reducing thermal load alongside restoring circulatory volume and electrolyte equilibrium. Oral rehydration remains a cornerstone for mild-to-moderate presentations, with fluid composition influencing rehydration efficiency. In this context, electrolyte-balanced formulations have gained attention for their ability to support rapid fluid uptake and physiological stability.

Recent developments highlight the role of ready-to-drink (RTD) hydration drinks as practical alternatives to conventional preparations. RTD formulations provide a pre-formulated combination of fluids and electrolytes in a ready-to-consume format, without the need for preparation or dilution. These offer advantages in consistency, ease of use, and acceptability, which may enhance adherence in real-world settings. Tailored strategies are particularly important for populations with heightened vulnerability to fluid imbalance, including older adults and individuals with comorbid conditions. Therefore, rehydration options, including RTD formulations, may provide a promising hydration-focused approach to mitigate heat exhaustion and improve clinical outcomes across diverse environmental and healthcare settings.

## Introduction and background

Rising temperatures are intensifying heat-related health risks globally and in India. Global mean temperatures have exceeded ~1.5°C above pre-industrial levels, reaching 1.62°C in 2024 [1,2]. Heat exhaustion, a common form of heat-related illness (HRI), has emerged as a significant and escalating health threat in the context of global climate change and is projected to increase by 40% to >100% by 2050 [3-5]. Between 2000 and 2019, heat illness-related mortality reached approximately 489,000 deaths annually worldwide, with Asia accounting for 45% [6,7].

In India, temperatures rose by 0.7°C during 1901-2018 and ~0.89°C during 2015-2024, with up to 4.4°C warming projected by century-end and 1.2-1.3°C by mid-century [8,9]. Future projection studies, including the India Meteorological Department reports, estimate an increase of ±1.2°C to ±3.5°C, with heatwaves becoming more frequent, intense, and long-lasting, potentially exacerbating chronic health conditions, vector-borne diseases, air pollution, and droughts, thereby increasing morbidity and mortality [10,11]. Between 1992 and 2015, 24,223 deaths were attributed to heatwaves, and heat-related mortality among older adults (≥65 years) rose by 55% between the 2000-2004 and 2018-2022 periods, and the 45-59 age group (men: 34.7%; women: 38.2%) bears the highest overall burden [11-13]. More than a billion Indians are now exposed to worsening heatwaves each year, with daytime temperatures regularly exceeding 40°C across states such as Delhi, Maharashtra, Uttar Pradesh, and the Indo-Gangetic plain. Geographic regions with prolonged heatwaves and urban areas with a heightened risk of the urban heat island phenomenon are especially prone to HRIs [14-16]. Vulnerable groups, including children, the elderly, pregnant women, outdoor workers, and individuals with chronic diseases, are most affected, particularly where healthcare access and cooling infrastructure are limited [3,17].

Despite this growing burden, most HRIs, including heat exhaustion, are preventable through timely and appropriate hydration [18]. Heat exhaustion is fundamentally linked to dehydration through the body's thermoregulatory response to elevated ambient temperatures [3]. The physiological cascade involves reduced cardiac output, compromised peripheral circulation, and diminished sweating capacity, all of which further impair the body's ability to maintain thermal homeostasis, producing intense thirst, sweating, weakness, headache, nausea, and muscle cramps [18,19]. Yet limited public awareness regarding the health risks of dehydration and a lack of understanding of optimal hydration practices during prolonged heat exposure continue to result in inadequate rehydration and poor clinical outcomes, highlighting the need for clearer, evidence-based guidance on prompt fluid and electrolyte replacement towards effective management of HRIs [3,20-23].

Recognizing the critical role of hydration in HRI, four multidisciplinary national experts in India convened to evaluate hydration drinks in scientifically formulated ready-to-drink (RTD) formats. The expert steering committee endorsed RTD isotonic hydration formulations based on their standardized electrolyte and energy composition, convenience, and enhanced palatability. Specifically, the experts recommended isotonic RTD hydration drinks containing key electrolytes - sodium, potassium, and chloride - with low sugar content for managing dehydration in nondiarrheal conditions, including HRIs. This narrative review explores the role of hydration in the prevention and management of heat exhaustion in different populations, with an emphasis on evidence-based strategies to mitigate dehydration-related complications, informed by expert guidance.

## Review

Literature search

A literature search was conducted to identify relevant evidence on HRIs in India, with particular emphasis on fluid-electrolyte balance and hydration management. Literature published between 1997 and 2026 was identified through searches of PubMed/MEDLINE and Google Scholar, along with relevant guidance and reports from the World Health Organization (WHO), India Meteorological Department (IMD), Ministry of Health and Family Welfare (MoHFW), National Centre for Vector Borne Diseases Control (NCVBDC), and other national and international health organizations. The search used combinations of key terms including “heat-related illness,” “heat exhaustion,” “heat stroke,” “heat stress,” “dehydration,” “electrolyte imbalance,” “fluid replacement,” “fluid-electrolyte management,” “oral hydration,” “oral rehydration,” “electrolyte drinks,” “isotonic,” “ready-to-drink,” “RTD,” “India,” and "Indian,” with relevant synonyms and combinations applied as appropriate. Clinical studies, reviews, guidelines, consensus recommendations, and authoritative reports addressing epidemiology, pathophysiology, clinical assessment, prevention, and management of HRIs, particularly fluid and electrolyte replacement, were considered. Priority was given to peer-reviewed clinical and epidemiological evidence, recent guidelines, and Indian data, while earlier publications were retained when they provided important foundational evidence. References were selected based on their relevance to the objectives and clinical context of the review.

Pathophysiology and classification of heat exhaustion

The pathophysiology of heat exhaustion involves a complex interplay between behavioral and autonomic responses aimed at maintaining normothermia [19]. Unlike normal thermoregulation via convection, conduction, radiation, and evaporation, when the ambient temperature exceeds core body temperature, the body's primary cooling mechanism shifts entirely to cutaneous vasodilation and evaporative heat loss through sweating. This can result in fluid losses exceeding 1.5 liters per hour during extreme heat exposure [3,19,24]. Additionally, a cytokine-mediated systemic inflammatory response also occurs upon sustained heat exposure [3]. Key mechanisms include increased cutaneous vasodilation for heat dissipation and enhanced sweating, which lead to significant fluid and electrolyte depletion, particularly of sodium and potassium. Dehydration and hypovolemia further impede cardiovascular function by diminishing central blood volume and cardiac output, resulting in symptoms such as weakness, dizziness, and fatigue [3,19,24]. If uncorrected, these pathophysiological cascades can progress to heat stroke, marked by systemic inflammation, hypervolemic circulatory shock, and multiorgan and central nervous system dysfunction (Figure 1) [18,19].

**Figure 1:**
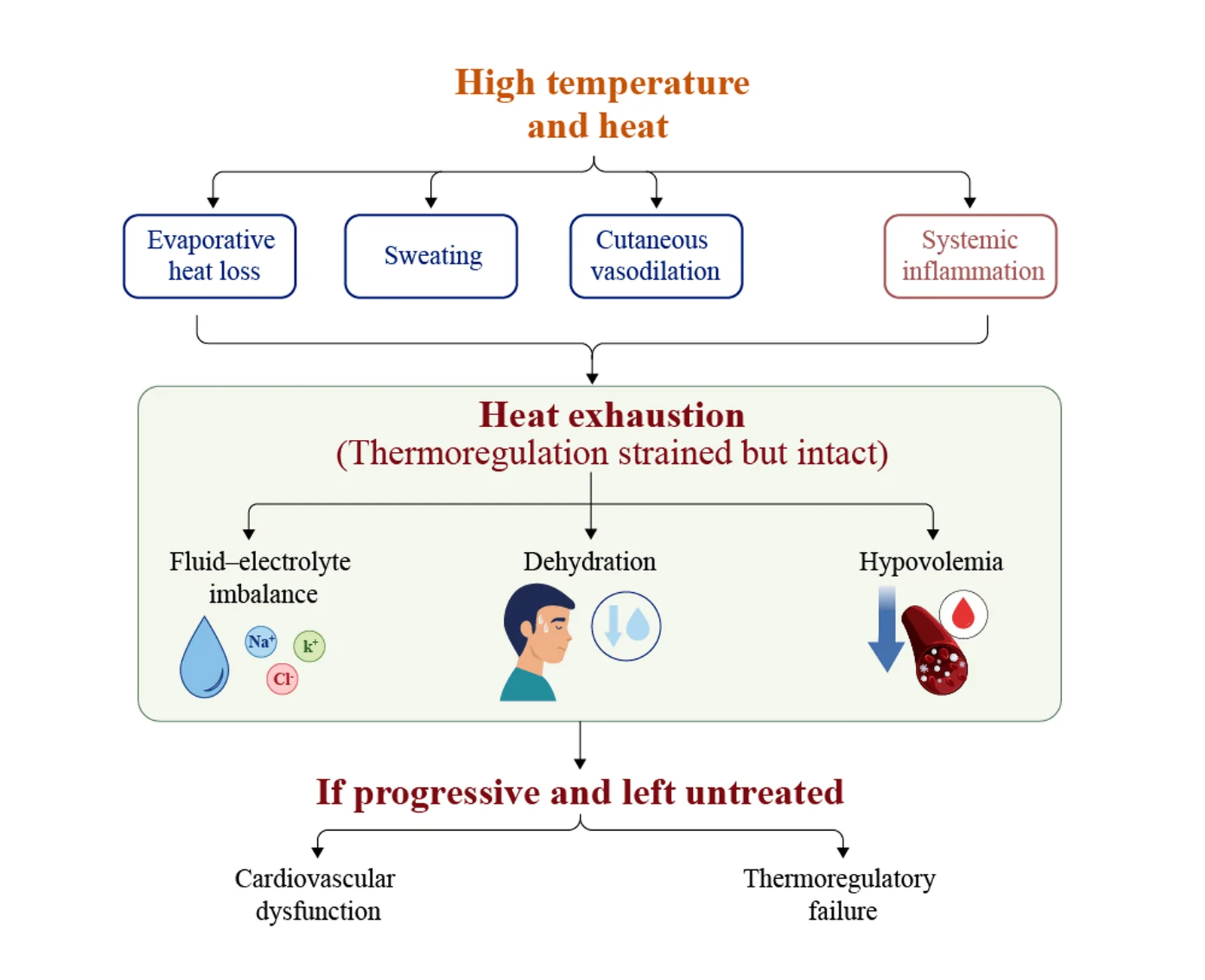
The pathophysiology of heat exhaustion. This original figure was created using Adobe Illustrator (Adobe Inc., San Jose, CA, USA), with free icons sourced from Magnific.

Heat exhaustion can be classified based on the underlying cause of fluid and electrolyte disturbance. Water-depletion heat exhaustion occurs when fluid intake is inadequate to compensate for excessive sweating, leading to severe dehydration with hypernatremia (serum sodium >145 mEq/L). This form presents with progressive hypovolemia, thirst, hyperthermia, and hyperosmolarity. Salt-depletion heat exhaustion develops in response to fluid replacement with hypotonic solutions lacking adequate electrolytes, resulting in hyponatremia (serum sodium <135 mEq/L) and subsequent cell swelling [18,19].

Clinical presentation and hydration status in heat exhaustion

Initial manifestations of heat exhaustion include increased thirst, headache, fatigue, malaise, and dizziness, which may progress to muscle cramps, confusion, and circulatory instability as core temperature rises beyond 38°C [18,24-26]. Fluid and electrolyte deficits are central to the majority of symptoms, linking dehydration and electrolyte imbalance to both muscular and neurological symptoms [25,27]. The serum electrolyte-environmental theory attributes muscle cramps to the loss of sodium, potassium, magnesium, chloride, and calcium through sweating or excessive water intake, linking it to heat-induced dehydration and electrolyte imbalance [28]. Additionally, earlier studies have associated higher temperatures and heat stress with hypoglycemia through increased vasodilation and insulin sensitivity. This further contributes to fatigue, weakness, and confusion, thus compounding the symptoms of heat exhaustion [29]. Moreover, fever or elevated core temperature may aggravate ongoing fluid and electrolyte losses by increasing metabolic rate and sweat volume, thereby intensifying dehydration. The interplay between hypovolemia, electrolyte loss, and thermoregulatory strain highlights the multifactorial etiology of heat exhaustion, demonstrating the pivotal role of hypoglycemia and fluid and electrolyte depletion in its clinical manifestation [25,26].

Approaches to heat exhaustion-related dehydration management

Clinical Management and Rehydration in HRIs

Heat exhaustion management requires prompt intervention to reduce core body temperature (at 0.20-0.35°C per minute) and restore fluid-electrolyte balance [30]. Dehydration is a key feature of heat exhaustion, making effective rehydration crucial for restoring and correcting plasma volume and electrolyte imbalances [3,18,24]. The Wilderness Medical Society guidelines advocate managing HRIs by immediately moving the patient to shade or air-conditioning, stopping physical activity, removing excess clothing, initiating cooling, providing fluid replacement, and monitoring [31,32]. Oral hydration with isotonic or hypertonic fluids, or intravenous (IV) administration of isotonic fluids, is recommended for mild-to-moderate HRIs, including heat exhaustion [31,32]. Importantly, the 2024 update explicitly cautions that heat stroke can occur irrespective of hydration status. Accordingly, fluid administration should be judicious, particularly in patients with cardiac comorbidities, to avoid pulmonary edema. Rehydration must not delay rapid whole-body cooling, which remains the priority intervention when HRI is suspected [32]. Fluid replacement strategies depend on the degree of dehydration, level of consciousness, tolerance for oral fluids, and any complicating factors [30,32]. The American College of Sports Medicine (ACSM) expert consensus on exertional heat illness emphasizes prompt recognition, rapid cooling, and timely medical assessment for reducing heat-related morbidity and mortality. Fluid replacement with oral fluids/hydration drinks is preferred over IV fluids and should therefore be initiated as soon as clinically feasible after successful cooling and recovery of consciousness [33]. The United States Military clinical practice guideline emphasizes prevention, early recognition, and rapid treatment of exertional heat illness. Key measures include risk assessment, heat acclimatization, hydration, and environmental monitoring. Rehydration should be individualized, avoiding both dehydration and overhydration, and cooling should not be delayed for fluid administration [34]. In addition, evidence-based exertional heat illness prevention centers on individualized hydration strategies that aim to prevent >2% body-weight loss while avoiding overhydration and hyponatremia. Hydration drinks are oral fluid-electrolyte solutions that are widely used to restore the fluids and electrolytes depleted during dehydration. They are pivotal to hydration management: water is generally adequate for exercise lasting <1 hour, while carbohydrate-electrolyte hydration drinks are recommended during prolonged activity. Following exercise, rapid rehydration with sodium-containing hydration drinks promotes recovery and fluid retention. Heat acclimatization and wet‑bulb globe temperature-guided activity modification reduce risk, whereas exertional heat stroke requires immediate recognition and aggressive cooling, preferably cold-water immersion followed by oral rehydration after stabilization [35].

India’s national action plan for HRI management adopts a spectrum-based approach. For heat exhaustion, this includes removal from the heat source, volume replacement, and oral or intravenous rehydration. In mild-to-moderate cases, oral rehydration with salt-containing fluids such as oral rehydration solution (ORS), lime water with salt and sugar, coconut water, or rice water is advised. Intravenous fluid therapy is indicated when oral intake is not tolerated. If there is no clinical improvement within 30 minutes of initial rehydration, aggressive cooling with continued resuscitation is warranted [36]. The National Centre for Disease Control (NCDC) recommends immediate removal from heat exposure, cessation of physical activity, and initiation of passive cooling using cool wet towels, ice packs, water spraying, and fanning. Patients with temperatures below 104°F should be reassessed and encouraged to take oral hydration drinks or ORS, while temperatures ≥104°F require intravenous rehydration and urgent hospital transfer [37]. The NDMA guidelines further emphasize adequate hydration as the cornerstone of prevention and early management of HRIs [38]. The international and national guidelines and recommendations for the management of HRIs are tabulated in Table 1. However, despite these broad recommendations, detailed India-specific clinical guidelines for comprehensive HRI management remain limited.

**Table 1:** Guideline for the management of HRI AC: air-condition; IV: intravenous; ORS: oral rehydration solution; RTD: ready-to-drink

S. No.	Source	Key recommendation
International guidelines		
1	Wilderness Medical Society (WMS) [31,32]	1. Immediate removal from heat (shade/AC), cessation of exertion, and removal of excess clothing 2. Start active cooling and assess hydration status promptly 3. Use oral isotonic/hypertonic fluids or IV isotonic fluids 4. Individualize fluid therapy based on clinical status, consciousness, and oral tolerance
2	American College of Sports Medicine (ACSM) [33]	1. Early recognition 2. Immediate cooling and prompt medical evaluation 3. Initiate oral rehydration as soon as feasible if the patient is conscious and able to tolerate fluids
3	United States Military Joint Service Medical Providers [34]	1. Early recognition 2. Prompt risk assessment, heat acclimatization, hydration planning, and environmental monitoring 3. Rapid cooling; must precede fluid administration 4. Individualized fluid replacement 5. Avoid both underhydration and overhydration
National (India-specific) guidelines		
4	National Action Plan for Heat Related Illnesses [36]	1. Stepwise management approach 2. Remove patient from heat and initiate fluid replacement 3. Use oral rehydration (ORS or salt-containing fluids) in mild–moderate cases 4. Administer IV fluids if oral intake is not feasible 5. Escalate to active/aggressive cooling with ongoing resuscitation if no improvement within 30 minutes
5	National Centre for Disease Control (NCDC) [37]	1. Immediate removal from heat and cessation of physical activity 2. Start passive cooling measures (wet towels, ice packs, water spray, fanning) 3. If temperature <104°F, give oral fluids/ORS and reassess 4. If temperature ≥104°F, start IV rehydration and arrange urgent hospital transfer
6	National Disaster Management Authority (NDMA) [38]	1. Prioritizes adequate hydration
7	Association of Physicians of India (API) [64]	1. Recommends RTD fluids for fluid-electrolyte supplementation 2. Supports RTD use during hospital care, IV-to-oral transition, and discharge
8	Research Society for the Study of Diabetes in India (RSSDI) [79]	1. RTD formulations combining fluids, electrolytes, and slow-release carbohydrates support balanced hydration and electrolyte replacement 2. Tailored fluid-electrolyte strategies may improve hydration, electrolyte balance, and glycemic control in clinical care

Evidence Supporting Oral Rehydration

Oral rehydration is preferred for mild-to-moderate dehydration due to its physiological advantages in fluid and electrolyte replenishment and lower complication risk. Oral fluid ingestion stimulates oropharyngeal receptors and a cascade of gastrointestinal hormones that regulate fluid distribution and thirst more physiologically compared to IV infusion, which bypasses these pathways [39]. Intravenous therapy is indicated in severe cases or when oral intake is not feasible. However, emergency medical care is necessary if symptoms persist, worsen, or neurological changes develop to prevent progression to heat stroke [30,32]. The Wilderness Medical Society recommends IV isotonic fluids should be initiated concurrently with conductive and convective cooling for severe heat exhaustion [32,39]. A randomized clinical trial comparing IV versus oral isotonic rehydration (with 0.45% NaCl) following exercise-induced dehydration of approximately 4% body weight demonstrated no statistically significant differences in exercise time to exhaustion (34.9 ± 4 min oral vs. 29.5 ± 3.5 min IV; p = 0.07), though perceptual measures including thirst ratings and perceived exertion were more favorable in the oral rehydration group [40]. In coherence, current evidence based on several studies concluded that IV rehydration confers transient advantages in the rate of plasma volume restoration, but these benefits are short-lived, and hence, do not support the routine use of IV over oral rehydration when the patient can tolerate oral intake [32,39,41-43].

Although data on rehydration specifically in heat exhaustion are limited, numerous studies have elucidated its critical role in the effective management of exercise- or work-related heat stress. An earlier report showed that oral rehydration improved fluid retention and recovery in a hot environment [44]. Furthermore, another study indicated that both intravenous and oral rehydration were effective in treating exercise-induced heat stress and dehydration during a 90-minute exercise session [45]. Public health advisories recommend staying hydrated by drinking water, using oral hydration drinks or homemade fluids like lemon water or buttermilk, and consuming water-rich fruits and vegetables such as watermelon, cucumber, and oranges to prevent HRIs during heat waves [46]. A randomized crossover study indicated that consuming oral hydration drinks during outdoor work in hot conditions reduced fatigue and the risk of HRI, including heat stroke, among outdoor workers [47]. Moreover, working in hot conditions can disturb homeostasis, leading to exertional HRI and fluid-electrolyte imbalances [48].

An emerging controversy concerns the role of the "drink to thirst" versus scheduled drinking strategy in heat-exposed individuals. Thirst is a reliable hydration guide for short-duration, lower-intensity exercise in cooler environments (<60-90 minutes), while programmed drinking to within ±2% body mass change is superior for prolonged exercise in the heat (>90 minutes), high sweat rates, and situations where performance is a concern [11]. It is recommended to avoid drinking to the point of weight gain, as this risks exercise-associated hyponatremia [49]. An Indian expert Delphi consensus on oral fluid and electrolyte management in adult and geriatric patients with nondiarrheal illness recommended oral fluid and electrolyte therapy as the core treatment strategy, advocating its early initiation and the use of appropriately formulated fluids with known electrolyte content to improve patient outcomes [50]. Collectively, these findings highlight that maintaining hydration with water and electrolyte-rich fluids is crucial for preserving thermoregulation, preventing HRI, and minimizing electrolyte imbalances during physical activity or environmental heat exposure.

Types of oral rehydration fluids and their physiological effects

Oral rehydration fluids are broadly classified into three categories based on their osmolality relative to plasma: hypertonic, hypotonic, and isotonic. Each has distinct physiological consequences for fluid absorption, electrolyte balance, and rehydration efficacy [51,52].

Hypertonic solutions, such as high-carbohydrate drinks, have an osmolality higher than plasma and are generally suboptimal for rapid rehydration. They can shift fluids and electrolytes from circulation into the intestinal lumen, transiently slowing absorption [51,53-55]. The Wilderness Medical Society recommends that sodium-rich hypertonic beverages (e.g., 3% sodium chloride) are beneficial in mild exercise-associated hyponatremia by increasing serum sodium concentrations and promoting symptom relief [56]. Hypotonic solutions, including World Health Organization oral rehydration solution (WHO-ORS), have a lower osmolality than plasma and are effective in promoting water absorption. The WHO-ORS, with an osmolarity of 245 mOsm/L, is effective for managing dehydration, as these formulations facilitate rapid fluid absorption while minimizing the risk of complications such as hyponatremia [57,58]. Therefore, it may be advisable for patients with heat exhaustion presenting with acute diarrhea. An earlier study shows that 10% glucose-electrolyte solutions (hypertonic) slow gastric emptying, delay plasma volume recovery, and retain gut fluid despite reduced urine output, while 2% solutions (hypotonic) empty faster and restore circulation more effectively, making hypertonic drinks unsuitable for rapid rehydration during short recovery periods [53]. However, hypotonic oral rehydration formulations are often associated with palatability challenges, which may limit voluntary intake and adherence in nonclinical settings or among the general population, despite the critical need for adequate hydration during prolonged heat exposure or physical exertion [31,32,46,59]. Additionally, consuming plain water in large volumes during prolonged physical activities is associated with an increased risk of exercise-associated hyponatremia. The Wilderness Medical Society clinical practice guidelines for exercise-associated hyponatremia management advocate fluid restriction and avoidance of oral and IV hypotonic fluids in fluid overload-associated exercise-associated hyponatremia and treatment of severe symptomatic cases with 3% hypertonic saline [56].

Isotonic fluids could be referred to as hydration or electrolyte drinks, which represent an optimal strategy for rehydration because their osmolality closely matches that of human plasma (≈275-300 mOsmol/kg), enabling rapid fluid absorption and effective cellular rehydration without inducing osmotic stress [52]. This physiological similarity minimizes osmotic gradients across the intestinal mucosa, thereby reducing the risk of fluid shifts into the intestinal lumen [51,52,60,61]. These formulations contain balanced concentrations of key electrolytes, particularly sodium, potassium, and chloride, along with appropriate amounts of carbohydrate. This composition enhances gastric emptying and intestinal uptake compared with plain water or hypertonic solutions [53,54]. Thus, replenishing electrolytes through isotonic hydration drinks or electrolyte drinks could serve the dual purpose of accelerating rehydration and restoring the ionic milieu necessary for efficient cellular energy production.

Clinical Benefits of Isotonic Hydration Drinks

Isotonic hydration drinks provide several advantages over standard ORS, conventional sports drinks, and plain water. In contrast to WHO-ORS, whose composition is strictly regulated and limits the inclusion of additional components, isotonic drinks allow greater formulation flexibility, enabling the incorporation of adjuncts such as taurine, calcium, and magnesium alongside optimally balanced electrolytes to support broader physiological demands during heat stress or physical exertion [62-64]. Sports drinks vary in composition and may contain suboptimal electrolyte concentrations, particularly sodium [65]. A randomized clinical trial demonstrated that beverages with higher sodium content (~60 mmol/L) during prolonged exercise in the heat resulted in a significantly greater plasma sodium concentration and attenuated plasma volume decline compared with a low-sodium formulation (~21 mmol/L), highlighting the importance of sodium in electrolyte-containing solutions [65]. Sodium is especially critical due to its role in the sodium-glucose cotransport mechanism (SGLT1) in the small intestine. The coupled absorption of sodium and glucose facilitates osmotic water movement, accelerating plasma volume expansion and rehydration [66]. Consequently, solute transport across the intestinal epithelium generates localized osmotic gradients that support net water uptake. These well-established transport mechanisms operate across various gastrointestinal conditions, contributing to the safety and tolerability of isotonic formulations [53,54,66]. Evidence suggests that each SGLT1 transporter is associated with the passive transport of 109 water molecules per second, highlighting its substantial contribution to intestinal water flux [67,68]. By preserving plasma tonicity, isotonic hydration drinks efficiently restore extracellular fluid and circulating volume [51]. Thus, standardized isotonic solutions (typically 35-70 mmol/L) could better align with physiological requirements for maintaining plasma sodium and volume during heat stress or prolonged exercise [62-64].

Beyond fluid balance, electrolytes in isotonic hydration drinks directly support cellular energy metabolism. Sodium and potassium gradients across cell membranes are maintained by the Na⁺/K⁺-ATPase, an enzyme that consumes a significant proportion of cellular ATP and is essential for nerve conduction, muscle contraction, and nutrient transport. Adequate availability of these ions ensures efficient ATP utilization and prevents early fatigue [69,70]. In addition, potassium is crucial for mitochondrial function and ATP synthesis, while magnesium acts as a required cofactor for ATP-dependent enzymes, stabilizes ATP molecules, and enables key steps in glycolysis and oxidative phosphorylation [71,72]. During heat exhaustion, electrolyte depletion impairs these metabolic processes, contributing to muscle weakness, cramping, and fatigue [18].

Isotonic formulations have also been shown to achieve hydration more rapidly, with evidence suggesting nearly two-fold faster rehydration compared to hypertonic fluids, further indicating their clinical advantage [60]. In the absence of electrolytes, plain water, when consumed in large volumes during significant dehydration, may dilute plasma sodium levels and slow effective rehydration, risking hyponatremia [51,53-55]. An earlier study confirmed that an isotonic beverage yielded a higher beverage hydration index (BHI) compared with distilled water, reflecting better fluid retention capacity compared to plain water [73]. Additionally, plasma volume expansion is greater with isotonic solutions containing adequate sodium (45 mEq/L) compared with plain water, despite marginally slower intestinal absorption kinetics relative to hypotonic formulations [52]. These findings support isotonic hydration drinks as a balanced and efficient rehydration strategy, offering effective electrolyte replacement, improved fluid retention, better palatability, and formulation versatility while overcoming the limitations associated with both hypotonic and hypertonic fluids.

Management of heat exhaustion-induced dehydration in high-risk patients

Elderly and Geriatrics

Dehydration in vulnerable populations, including older adults, should be regarded as a chronic condition necessitating proactive prevention through an individualized approach, encompassing individualized fluid intake goals, public health initiatives, community outreach, and clinical interventions [74]. In the geriatric population, physiological changes such as reduced thirst and diluted urine, accompanied by changes in water and nutrient absorption, increase susceptibility to fluid deficits [75,76]. A recent systematic review indicated that aging is associated with impairments in sweating, cutaneous vasodilation, cardiovascular adaptation, and hydration status in older adults, which increase their susceptibility to heat strain, dehydration, and fatigue [77]. Educating patients to recognize early signs of dehydration and maintain adequate fluid intake is crucial for preventing HRI. Older adults, in particular, are advised to drink ORS (240 mL/hour) during periods of high temperatures while limiting diuretic beverages such as coffee, tea, and alcohol [75,76]. A case report documented successful oral rehydration therapy in a 68-year-old at high risk of IV fluid overload, indicating that oral rehydration is a practical, low-cost alternative with the potential to circumvent IV-related complications [78].

Patients With Diabetes

Patients with diabetes face an increased risk of dehydration due to osmotic diuresis, especially in hot temperatures. This exacerbates hyperglycemia, leading to electrolyte imbalance and renal impairment, necessitating proactive hydration to prevent polyuria and preserve renal function, especially in patients with diabetic nephropathy [79]. Approaches to dehydration management in these patients primarily focus on maintaining adequate hydration, optimizing glycemic control, and preventing complications associated with excessive fluid loss [80]. Additional preventive strategies include regular blood glucose monitoring, medication review (particularly diuretics), maintaining hydration during hot weather and physical activity, and recognizing early symptoms of dehydration or heat exhaustion such as dizziness, excessive sweating, muscle cramps, headache, and fatigue [79].

According to the Research Society for the Study of Diabetes in India (RSSDI), plain water alone may be insufficient during dehydration associated with non-diarrheal illnesses, including HRIs. Early oral rehydration with electrolyte-containing beverages is recommended, preferably formulations containing optimal sodium and glucose concentrations to enhance water absorption. For people with diabetes, RTD electrolyte drinks containing low-glycemic, slow-release carbohydrates (e.g., isomaltulose, D-tagatose, or trehalose) are preferred, as they support hydration, replenish electrolytes, provide sustained energy, and minimize glycemic excursions [79]. A pharmacological concern involves sodium-glucose cotransporter-2 (SGLT2) inhibitors, which are increasingly prescribed for type 2 diabetes and heart failure. These agents promote glucosuria-driven diuresis, predisposing to volume depletion, orthostatic hypotension, and electrolyte imbalance, particularly when combined with concurrent diuretic therapy [81]. Hence, patients receiving SGLT2 inhibitors should increase daily water intake by 2-3 additional glasses to compensate for medication-induced diuresis. In low-resource settings, affordable alternatives such as ORS, buttermilk, coconut water, lightly salted lemon water, rice water, and water-rich fruits and vegetables may help maintain hydration and electrolyte balance when commercial formulations are unavailable [79]. The American Diabetes Association (ADA) guidelines also recommend that individuals with diabetes reduce their intake of both sugar-sweetened and non-nutritive sweetened beverages and opt for alternative options, particularly by increasing water consumption [80].

General Consideration

The route and rate of rehydration should be guided by the severity of dehydration, clinical parameters, and individual comorbidities. The Wilderness Medical Society practice guidelines note that for high-risk patients, including the elderly, obese individuals, or those with comorbidities such as diabetes, hypertension, and cardiovascular disease, evaporative-convective cooling methods, encompassing skin wetting and fanning, may have a supportive role in hospital-based management. However, these methods provide slower cooling than conductive techniques and are not recommended for exertional heat stroke [31]. Guidelines from the Indian Council of Medical Research-National Institute of Nutrition (ICMR-NIN) and the European Food Safety Authority (EFSA) emphasize frequent and adequate water intake to avoid dehydration [80,82,83]. Therefore, early identification of dehydration symptoms, followed by prompt and tailored rehydration interventions, is crucial for reducing the risk of progression to heat stroke and improving recovery outcomes.

Role of RTD electrolyte/hydration drinks in the clinical management of heat exhaustion

Recommendations for RTD Electrolytes/Hydration Drinks in HRI

Oral rehydration therapy (ORT) is well-established as a first-line intervention for managing dehydration and electrolyte imbalance in clinical practice [84], and it is specifically recommended as a first-line treatment for dehydration due to heat exhaustion. ORT operates through the sodium-glucose cotransport mechanism in the small intestine, enabling efficient absorption of water and electrolytes. This mechanism makes ORT superior to plain water for rehydration [85]. Commercially available oral ready-to-drink beverages are commonly referred to as hydration drinks or electrolyte drinks and are formulated to replenish body fluids lost during dehydration. These hydration or electrolyte drinks consist of hydration salts like sodium chloride, potassium chloride, and sodium citrate, reflecting their essential role in restoring the body's fluid and electrolyte balance and facilitating hydration during states of dehydration.

A study by Kanda et al. reveals that ORT alone may not be sufficient for severe cases of heat exhaustion. Active cooling methods have been shown to significantly lower mortality rates and improve overall outcomes in such cases [86]. This highlights the need for a multifaceted approach to treating heat exhaustion.

The API expert consensus recommendations mention the requirement of appropriate fluid and electrolyte supplementation using RTD fluids in patients with non-diarrheal illnesses, including HRIs, to support recovery during hospitalization, transition from intravenous to oral therapy, and at discharge. RTD formulations provide known concentrations of electrolytes, enabling standardized rehydration while improving palatability and reducing taste fatigue [64]. The RSSDI consensus further highlights the utility of RTD oral fluid and electrolyte drinks for individuals experiencing HRIs. Compared with homemade fluids, fruit juices, or coconut water, these formulations offer a more consistent and evidence-based approach to replenishing fluid and electrolyte losses (Table 1) [79]. Collectively, expert recommendations suggest that RTD electrolyte/hydration drinks can serve as a practical adjunct in the management of heat exhaustion by promoting effective rehydration, electrolyte replacement, sustained energy delivery, and recovery.

Advantages of RTD/Hydration Fluid-Electrolyte Drinks

RTD hydration drinks offer several advantages over conventional powdered oral hydration formulations in managing heat exhaustion, particularly for rapid, safe rehydration in hot, humid environments. RTD hydration drinks are hygienic, premixed, and eliminate preparation errors common with powdered formulations, such as incorrect glucose-to-sodium ratios that may cause suboptimal absorption or osmotic diarrhea [59]. These noncaffeinated, noncarbonated drinks are enriched with essential electrolytes and minerals such as calcium and magnesium, along with taurine (an amino sulfonic acid), all of which are crucial for muscle function and thermoregulation [50]. Calcium and magnesium support muscle contraction-relaxation balance and reduce the risk of heat-induced muscle fatigue [64]. Taurine, a conditionally essential amino acid, plays a vital role in electrolyte balance and osmoregulation within the body, especially during heat stress and dehydration. It acts as an intracellular osmolyte, regulates fluid movement, and helps cells maintain proper hydration status, which in turn impacts the concentration and distribution of electrolytes [62]. By functioning as an osmolyte, taurine ensures cellular volume regulation, preventing excessive electrolyte loss or accumulation, especially in muscle tissues and the brain [63].

Maintaining adequate hydration is critical in heat exhaustion, and the palatability of hydration drinks plays an important role in improving fluid intake and patient adherence. Evidence indicates that improved taste enhances voluntary fluid consumption and promotes effective rehydration, particularly during dehydration and heat stress [87-90]. The addition of sucralose to oral electrolyte drinks enhances taste without adding caloric or glycemic load. Approved by the WHO and the US Food and Drug Administration (FDA) and reaffirmed as safe by the European Food Safety Authority (EFSA) and the Joint FAO/WHO Expert Committee on Food Additives (JECFA), sucralose has been established as a safe nonsugar sweetener across population groups when consumed within authorized levels (acceptable daily intake: 15 mg/kg body weight per day) [91-94]. The inclusion of sucralose in RTD hydration drinks can facilitate better fluid consumption, thereby aiding timely correction of dehydration and improving clinical outcomes in HRIs.

Beyond their therapeutic role, RTD hydration drinks hold significant potential as a preventive strategy against HRIs, particularly in vulnerable populations such as outdoor workers, athletes, military personnel, and the elderly. Maintaining adequate prehydration and sustaining electrolyte balance before and during heat exposure is a well-recognized strategy for reducing the incidence and severity of HRIs [21]. Unlike plain water, RTD hydration drinks help replenish sodium and other electrolytes lost through perspiration, thereby supporting the maintenance of fluid balance, thermoregulation, and physical performance. In addition, the sodium content in RTD hydration drinks may help maintain hydration by stimulating thirst, improving fluid retention, and reducing the risk of dilutional hyponatremia associated with excessive plain water intake during prolonged heat exposure or exertion [95,96]. Furthermore, the presence of magnesium in these formulations may aid in the prevention of HRIs, as magnesium loss through sweat has been linked to heat cramps and neuromuscular dysfunction, and its replenishment may help reduce early manifestations of heat stress [97]. Notably, the portability, palatability, and ease of use of RTD hydration drinks, due to their convenient, ready-to-consume format, may reduce barriers to adequate hydration compliance in field and occupational settings. This makes them a practical public health tool in heatwave preparedness, especially in a country like India where heatwave frequency and intensity are projected to increase substantially in the coming decades [8,9,11].

Knowledge gaps and future directions

Oral rehydration with isotonic electrolyte/hydration drinks, preferably in RTD formulations, constitutes the cornerstone of mild-to-moderate heat exhaustion management. Current evidence and clinical guidance consistently support early fluid and electrolyte replacement; however, several knowledge gaps remain [31-34,36-38,64,79]. Guidelines and clinical evidence, including Wilderness Medical Society updates and comparative rehydration trials, robustly support early oral rehydration for mild-to-moderate cases, reserving intravenous therapy for severe presentations, and combining active cooling with rehydration in refractory cases [30-32,39,40,86].

In contrast, much of the physiological basis for isotonic formulation superiority, osmolality-based fluid classification, and roles of specific electrolytes (sodium, potassium, magnesium, taurine) derives from exercise physiology studies in athletes rather than clinically diagnosed heat exhaustion patients and has been interpreted as extrapolated and mechanistically supported evidence rather than direct clinical proof [52-54,65-73]. Additionally, recommendations for RTD hydration drinks in high-risk and vulnerable populations, including older adults, individuals with diabetes mellitus, patients on polypharmacy, outdoor workers, and those in resource-constrained settings, are mostly expert consensus, such as the API and RSSDI Delphi panels [64,75,77-79]. Notably, there is a lack of large randomized controlled trials evaluating important clinical outcomes such as symptom resolution, hospital admission, progression to heat stroke, length of stay, and mortality in patients with clinically diagnosed heat exhaustion. For Indian healthcare settings, the applicability of these recommendations is supported by national guidance from the NDMA, ICMR-NIN, and the National Programme on Climate Change and Human Health, which directly addresses local climatic conditions and occupational heat exposure. However, cautious extrapolation is warranted where the underlying physiological evidence is derived primarily from temperate-climate or exercise-based cohorts [36,38,82]. Moreover, prospective data from India on dehydration patterns, electrolyte disturbances, and rehydration outcomes in primary care and occupational health settings are scarce. Clinicians should prioritize guideline-supported core interventions while treating formulation- and population-specific claims as adjunctive. Future research should focus on pragmatic clinical trials comparing oral rehydration strategies, assessing cost-effectiveness, and identifying optimal formulation characteristics for improvised management of HRIs.

## Conclusions

Heat exhaustion is an increasing health concern in India, where extreme heat and humidity increase the risk of fluid-electrolyte losses and progression to potentially life-threatening heat stroke. Effective management requires a dual approach: immediate cooling combined with rapid restoration of fluid-electrolyte balance, preferably using RTD hydration drinks, through evidence-based rehydration strategies. Optimal formulations should include potassium, magnesium, calcium, and taurine. These electrolytes and osmolytes prevent cramping, maintain neuromuscular function and muscle contraction-relaxation balance, reduce heat-induced fatigue, and support cellular hydration during heat stress. However, the choice of rehydration approach should be individualized and tailored to the clinical severity of HRI, hydration status, and individual patient characteristics. Therefore, as the HRI burden rises, strengthening heat-exhaustion-specific clinical evidence is essential to refine rehydration strategies, inform population-specific recommendations, and improve preparedness and outcomes in heat-vulnerable settings.
